# Development of a Prediction Model for Progression Risk in High‐Grade Gliomas Based on Habitat Radiomics and Pathomics

**DOI:** 10.1002/acn3.70304

**Published:** 2026-01-04

**Authors:** Yuchen Zhu, Yuxi Gong, Weilin Xu, Xingjian Sun, Gefei Jiang, Lei Qiu, Kexin Shi, Mengxing Wu, Yinjiao Fei, Jinling Yuan, Jinyan Luo, Yurong Li, Yuandong Cao, Minhong Pan, Shu Zhou

**Affiliations:** ^1^ Department of Radiation Oncology The First Affiliated Hospital of Nanjing Medical University Nanjing Jiangsu China; ^2^ The First School of Clinical Medicine Nanjing Medical University Nanjing Jiangsu China; ^3^ Department of Pathology The First Affiliated Hospital of Nanjing Medical University Nanjing Jiangsu China; ^4^ Second Affiliated Hospital Zhejiang University, School of Medicine Zhejiang Hangzhou China

**Keywords:** habitat radiomics, high‐grade gliomas, pathomics, prediction model, progression risk

## Abstract

**Objective:**

To investigate the value of constructing models based on habitat radiomics and pathomics for predicting the risk of progression in high‐grade gliomas.

**Methods:**

This study conducted a retrospective analysis of preoperative magnetic resonance (MR) images and pathological sections from 72 patients diagnosed with high‐grade gliomas (52 cases as a train cohort and 20 cases as a test cohort). The regions of interest (ROIs) were annotated accordingly. In MRI processing, the ROI was further divided into clusters to extract habitat radiomics features. For whole slide imaging (WSI), the ROI was cropped into equal‐sized image patches for weakly supervised learning and deep learning using various network architectures. The optimal model architecture was selected, and pathological features were extracted. After feature selection, four independent models were constructed: habitat radiomics model, pathomics‐based model, clinical model, and combined model integrating all information. Model performance was evaluated using the concordance index (C‐index) and the area under the receiver operating characteristic curve (AUC).

**Results:**

The combined model demonstrated the best predictive performance, with a C‐index of 0.883 and an AUC of 0.965 in the train cohort. In the test cohort, the C‐index was 0.840, and the AUC was 0.927. Based on the combined model, patients with high‐grade gliomas were divided into high‐risk and low‐risk groups, with median progression‐free survival (mPFS) of 9 months and 77 months, respectively (*p* < 0.001).

**Conclusion:**

Compared with the habitat radiomics model or the pathomics‐based model alone, the combined model can better predict the risk of progression in high‐grade gliomas and provides valuable guidance for personalized treatment of high‐grade gliomas.

## 1 Introduction

According to a survey by the Chinese Society of Oncology, brain gliomas have an annual incidence of 6.4 per 100,000, making them the most common primary malignant tumor in the adult central nervous system. The current WHO glioma grading criteria integrate three aspects, including histological features that evaluate the differentiation of indicator cells (astrocytes, oligodendrocytes, or ependymal cells) and tumor type, histological features of anaplasia indicating the WHO grade of the central nervous system, and the results of diagnostic molecular marker detection [1]. High‐grade gliomas (HGG, grade 3–4) account for about 74.2% of gliomas [2, 3]. The primary treatments for HGG include maximal surgical resection, radiotherapy, temozolomide chemotherapy, and tumor‐treating fields [4, 5, 6]. However, the 5‐year overall survival rate remains low, between 6.6% and 30.9%. Research indicates that patients with disease progression within 1 year have a poorer prognosis [7, 8, 9]. Identifying patients with progression within the year before treatment is crucial for personalized interventions and improving survival rates.

“Radiomics” is an emerging field that transforms medical images into high‐dimensional, quantitative features through data extraction algorithms. These features have prognostic value for predicting clinical outcomes in various cancers and assessing treatment responses [10, 11]. Based on radiomics, habitat radiomics analysis quantifies tumor heterogeneity to identify tumor subregions or “habitats” with imaging biomarker characteristics [12, 13]. By clustering similar voxels, it determines tumor heterogeneity and quantifies significant tissue changes after treatment [14, 15]. This approach has demonstrated predictive potential across various cancers, including nasopharyngeal carcinoma and lung cancer [12, 13, 16].

Histopathological examination of tissue sections is critical for cancer diagnosis and treatment planning, providing high‐resolution images of tumor morphological features [17, 18]. However, this technique relies heavily on pathologist's expertise [19]. Pathomics, Pathomics aims to extract information from digitized high‐resolution whole‐slide images (WSIs) to obtain quantitative data, addressing these issues and offering valuable scientific and clinical insights [20, 21, 22]. In the era of artificial intelligence in oncology, deep learning‐based pathomics can identify complex patterns and biological characteristics associated with risk, enabling prognostic predictions. It has been applied in nasopharyngeal carcinoma and gastric cancer [23, 24]. However, the predictive effect of single omics on cancer can sometimes be inadequate.

Radiopathomics connects radiomics and pathomics, offering a multidimensional perspective for studying tumor heterogeneity at both macro and micro scales, often termed “virtual biopsy” [25, 26]. Radiomics captures subtle changes within tumors, revealing macro‐level heterogeneity to enhance predictive models [27, 28]. Pathomics analyzes histopathological images, providing data on micro‐localized lesions [27, 29]. Since radiological and pathological data complement each other regarding tumor biology, their integration may improve tumor characterization and lead to more robust models [27]. This approach has been applied to prognostic predictions in tumors like nasopharyngeal carcinoma, hepatocellular carcinoma, and glioblastoma [25, 26, 27, 29]. However, combining habitat radiomics with pathomics for prognosis prediction in high‐grade gliomas has not been extensively studied.

The objective of this study is to develop an appropriate model using habitat radiomics analysis and pathomics to predict the progression risk of patients with high‐grade gliomas and to provide insights and guidance for personalized treatment of patients.

## 2 Methods

### 2.1 Patients

**FIGURE 1:**
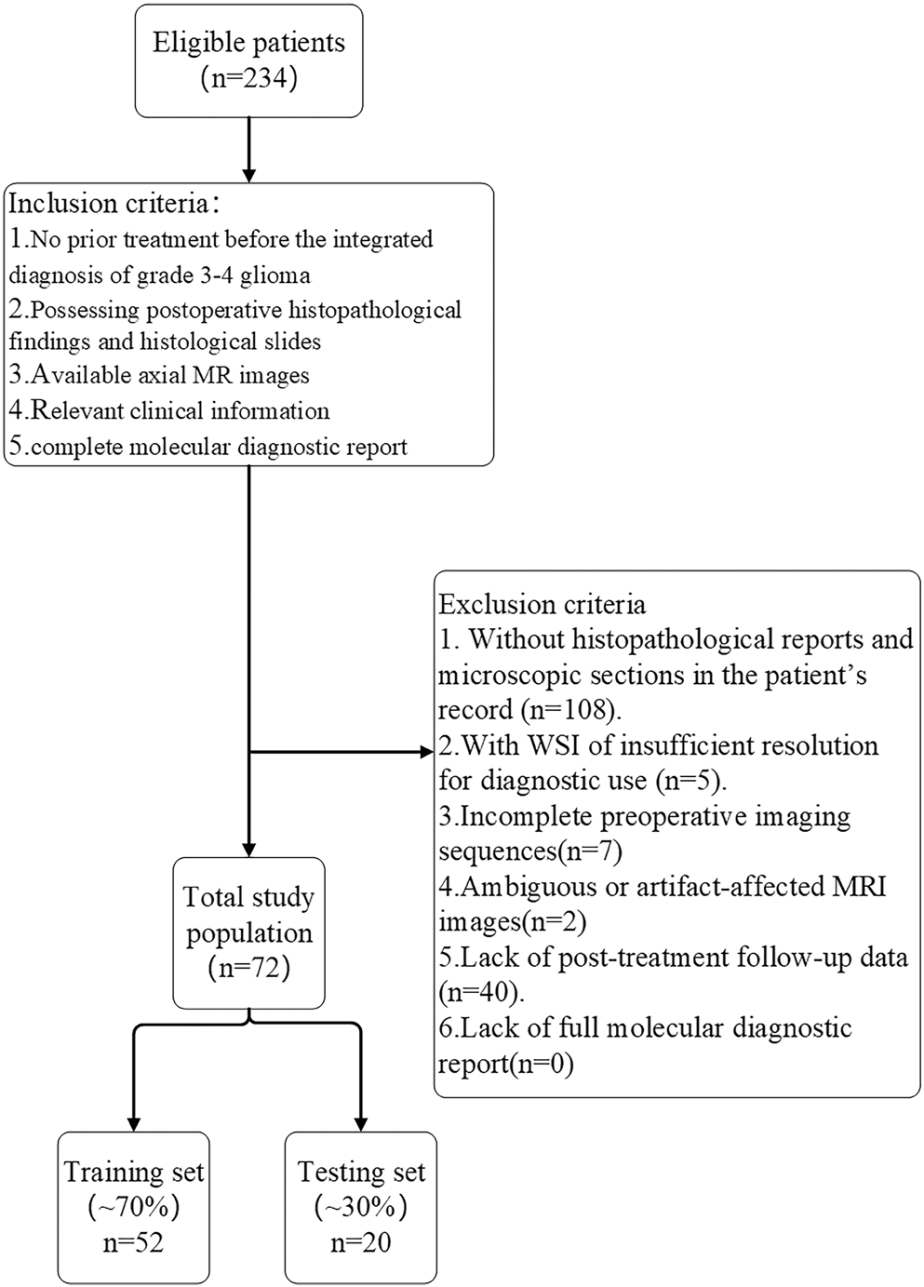
Flow chart of patient selection.

Between March 2015 and June 2023, we consecutively included patients with high‐grade gliomas diagnosed at our center using comprehensive pathology and molecular biomarkers. For this retrospective analysis, inclusion criteria required patients to have: (1) No prior treatment before the integrated diagnosis of grade 3–4 glioma, (2) Possessing postoperative histopathological findings and histological slides, (3) Available axial MR images, (4) Relevant clinical information, and (5) Complete molecular diagnostic report. Exclusion criteria included patients with: (1) Without histopathological reports and microscopic sections in the patient's record, (2) With WSI of insufficient resolution for diagnostic use, (3) Incomplete preoperative imaging sequences, (4) Ambiguous or artifact‐affected MRI images, and (5) Lack of post‐treatment follow‐up data. The flowchart of patient selection is depicted in Figure 1. The study has been approved by the Ethics Committee of the First Affiliated Hospital of Nanjing Medical University (No. 2024‐SR‐913). Since this is a retrospective study, informed consent was waived.

### 2.2 Treatment, Follow‐Up, and Clinical Endpoint

Patients who met the eligibility criteria received a combination treatment approach. This involved radiotherapy delivered at a total dose of 60 Gy over a span of 6 weeks, divided into 30 fractions. Concurrently, temozolomide was administered daily at a dosage of 75 mg/m^2^ for 6 weeks, followed by a 4‐week treatment break. Subsequently, patients underwent maintenance therapy consisting of temozolomide at a daily dosage of 150–200 mg/m^2^ on days 1–5 within each 28‐day cycle, for a maximum of 6 cycles.

Approximately 3 months after treatment, patients underwent dynamic observation before undergoing MRI and functional MR imaging. Recurrence assessment followed the RANO [30, 31] (Response Assessment in Neuro‐Oncology) standard and was conducted by a multidisciplinary team (MDT) comprising experts from the Radiotherapy Department, Neurosurgery Department, and Radiology Department. The MDT comprehensively analyzed clinical manifestations, scope of enhancement, and timing of recurrence for each patient. MRI scans were performed for all patients, and the need for additional functional MRI techniques such as magnetic resonance spectroscopy (MRS) and perfusion‐weighted imaging (PWI) was evaluated to aid in diagnosis. Disease progression was determined based on the following criteria: (1) Target lesions: ≥ 25% increase in sum of the products of perpendicular diameters, or ≥ 40% increase in total volume of enhancing or nonenhancing target lesions, or both, on stable or increasing doses of corticosteroids not attributable to radiation effect, edema, or comorbid events (2) New enhancing or nonenhancing lesion: In the case where the baseline or best response demonstrates nomeasurable disease (visible or not visible), then any new measurable (> 10 mm × 10 mm) lesions are considered PD [32]. The primary endpoint of the study was progression‐free survival (PFS), defined as the duration during which patients showed no signs of disease progression either during or after treatment.

### 2.3 Clinical Data Acquisition

Before treatment, we collected clinical characteristics from our health information system (HIS). These included demographic parameters such as age, sex, height, and weight, as well as clinical variables like chronic disease history, family medical history, glioma grade, body mass index (BMI), glioma type, and so on. Tumor measurements, including volume, were obtained by defining the ROI using ITKSNAP software.

### 2.4 Study Design

**FIGURE 2:**
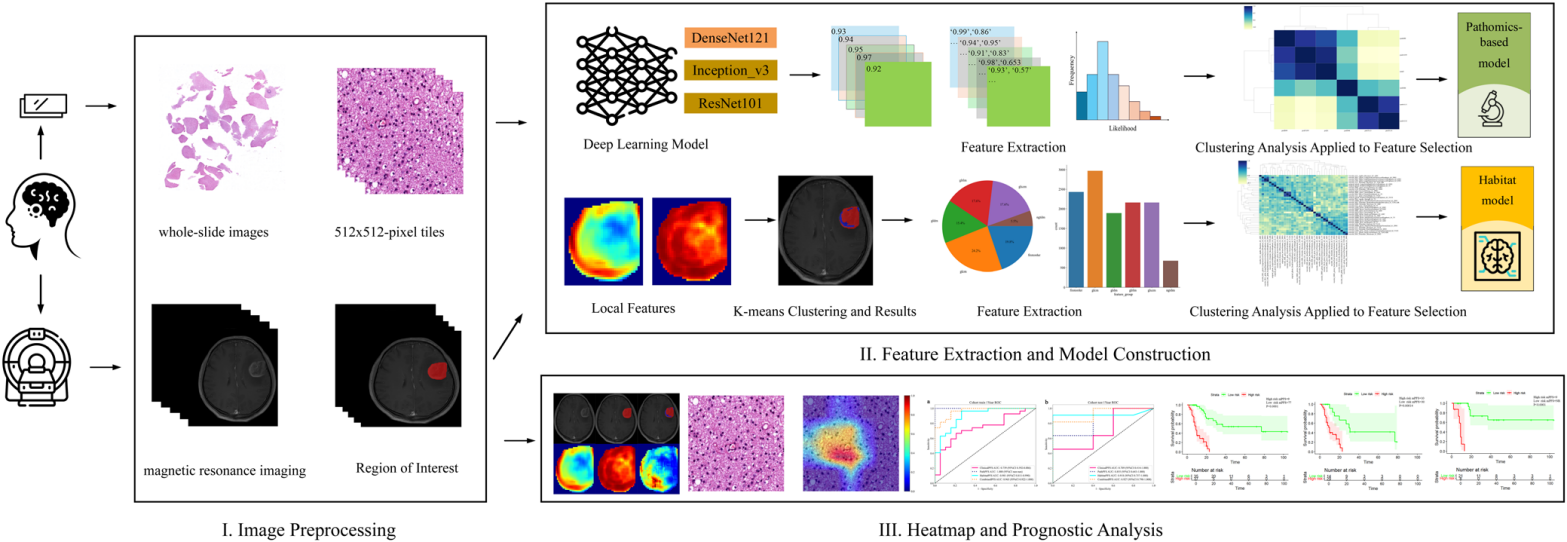
The workflow of the glioma habitat and histopathological signature assessment method used in this study.

A retrospective cohort design was employed to analyze MRI data involving multiple imaging sequences and WSI collected from a single institution. The relevant flowchart is shown in Figure 2.

For the multi‐sequence MRI data, the initial phase included image preprocessing techniques such as delineating the ROI, registration, and normalization tasks. Subsequently, the K‐means clustering method was employed to segment the regions within the tumor into different habitats and extract their features. In the final step, habitat radiomics features were selected using Pearson correlation analysis, univariate Cox regression, and LASSO Cox regression analysis.

For the WSI data, the initial phase involved image preprocessing techniques, including delineating the ROI, cropping the WSI into patches, removing blank areas, and normalization. Subsequently, the cropped patches were trained using various architectures based on weakly supervised learning. The trained deep model is then used to predict the label for each patch, which generates patch‐level predictions and combines probability histograms and TF‐IDF features to form pathological features. In the final step, pathological features were selected using Pearson correlation analysis, univariate Cox regression, and LASSO Cox regression analysis. The detailed flowchart can be seen in Figure 2.

### 2.5 Imaging Segmentation

Our dataset includes five imaging sequences: ADC, DWI, T1CE, T1W, and T2‐FLAIR. All patients underwent 3.0‐T MRI scans that covered T1‐weighted (T1W), T2‐weighted Fluid‐Attenuated Inversion Recovery (T2‐FLAIR), diffusion‐weighted imaging (DWI), apparent diffusion coefficient (ADC), and T1‐weighted contrast‐enhanced imaging (T1CE). The parameters for these sequences can be found in Data S1.

### 2.6 MRI Data Processing and Model Construction

In our medical image analysis study, we adopted a standardized approach to achieve consistent voxel spacing, utilizing a fixed resolution resampling strategy to standardize the resolution of varied volumes of interest to 1 mm × 1 mm × 1 mm. This standardization was essential for enabling accurate image comparisons and significantly improving the precision and reliability of our analysis results.

Our dataset includes five distinct imaging sequences: ADC, DWI, T1C, T1, and T2FLAIR. For the T1CE sequence, two experienced radiologists independently delineated the Region of Interest (ROI), which includes the enhanced area, non‐enhanced area, and necrotic area, excluding the abnormal signals surrounding the enhanced area, using ITK‐SNAP software [33, 34]. In cases where discrepancies arose between their annotations, an expert radiologist with 20 years of experience was consulted to resolve the differences. Examples of relevant ROI delineation can be found in Figure S1. For the remaining sequences, we employed automatic registration to the T1CE sequence via ITK‐SNAP, followed by meticulous adjustments made by professional radiologists to ensure optimal alignment.

We tackled the challenge of manually outlining tumor subregions in high‐grade gliomas by implementing an automatic segmentation method using the K‐means clustering algorithm. This approach effectively partitioned the tumor into distinct regions, each exhibiting uniform signal intensity across various MRI modalities, including T1CE, ADC, DWI, T1W, and T2‐FLAIR.

To analyze local features such as entropy and energy for precise characterization of ROI regions, we applied a 5 × 5 × 5 moving window on T1CE images. This process generated a 19‐dimensional feature vector for each voxel, as detailed in Data S2. By integrating these local features with voxel intensities from all modalities, we constructed a 24‐dimensional global matrix for each sample. This matrix was subsequently processed using voxel‐based K‐means clustering to delineate the tumor into distinct habitats, as outlined in Data S3 and Equation (A.1) in Data S1.

The automated segmentation algorithm successfully produced spatially distinct habitats characterized by homogeneous signal patterns. To determine the optimal number of clusters, ranging from 2 to 10, we evaluated various metrics—including the Calinski–Harabasz score, the Silhouette score, and the Davies–Bouldin index—using the scikit‐learn package.

For a comprehensive analysis of each subregion across all modalities, we categorized the features into Geometry, Intensity, and Texture, extracting them with Pyradiomics 3.0.1 software according to established guidelines. The extraction process was conducted independently for each subregion, and when regions exhibited insufficient voxel counts, we employed a k‐nearest neighbors strategy.

To filter out redundant features, we applied Pearson's correlation analysis, selecting only those features with a correlation coefficient below 0.9. We further refined feature selection through univariable Cox regression, which ranked features based on their *p*‐values. The final feature set was determined using LASSO‐Cox regression, employing cross‐validation methods to identify the optimal regularization parameter. Additional details on this process can be found in Data S4.

Finally, after conducting LASSO feature screening, we utilized Cox regression to model the selected features and estimate the average expected survival time, leading to the development of our habitat signature. It is important to note that the habitat signature, derived from the habitat radiomics analysis results, offers a unique perspective for understanding the complexities of intratumoral heterogeneity.

### 2.7 WSI Data Processing and Model Construction

In this study, the Hamamatsu digital whole slide scanner NanoZoomer S360 was used to scan the pathological tissue sections into whole‐slide images (WSI). The regions of interest (ROI) in the WSI of our dataset were independently delineated by two experienced pathologists using QuPath software. In cases where discrepancies existed in their annotations, these were resolved by a senior pathologist with 18 years of experience. Subsequently, further processing involved segmenting them into 512 × 512‐pixel tiles at 20× magnification to effectively manage their large size. During this process, we removed all white backgrounds to eliminate tiles with sparse informational content, specifically those containing predominantly bright pixels. This refined selection process resulted in over 12 million viable patches. All preprocessing tasks were performed on the OnekeyAI Platform, utilizing the OKT‐crop_WSI2patch tool for cropping, OKT‐patch2predict for removing white backgrounds, and OKT‐patch_normalize for standardizing color appearance.

The deep learning pipeline we use includes a dual‐tier prediction framework that merges patch‐level predictions with multi‐instance learning to compile features from whole slide images (WSI). During the training phase, we adopted a weakly supervised learning approach, where patches were consistently labeled according to the 1‐year recurrence of the associated patient, which served as the training label. The densenet121, inception_v3, and resnet101 architectures were utilized for training these patches. For a comprehensive description of the model structure, please refer to Data S5.

Following the completion of our deep learning model's training, we predicted labels and their corresponding probabilities for individual patches. The probabilities were subsequently merged using a classifier, resulting in predictions for the entire slide image (WSI). For more information, please consult Data S6.

In this study, we utilized a radiomics‐like methodology to develop the histopathological signature, combining patch‐level predictions, probability histograms, and TF‐IDF features into a unified feature set. To identify and remove redundant features, we utilized Pearson's correlation analysis [35], selecting only those with a correlation coefficient below 0.9. Feature selection was further refined by employing univariable Cox regression, which ranked features based on their *p*‐values. The final set of features was determined using LASSO‐Cox regression, and cross‐test methods were applied to identify the optimal regularization parameter. Data S4 provides additional details on this process.

Following Lasso feature screening, Cox regression was employed to model the selected features and estimate the average expected survival time, resulting in the development of our histopathological signature.

### 2.8 Combined Model Construction, Evaluation, and Survival Analysis

Univariable Cox regression and LASSO‐Cox regression were carried out to identify a clinical signature with no significant difference between groups from all clinical parameters. A clinical model was constructed by clinical signature with no significant difference between groups. Subsequently, a habitat model and a pathomics‐based model were constructed through habitat signature and histopathological signature, respectively, and habitat, histopathological, and clinical signatures were integrated to build a combined model. The specific model construction method and cross‐validation are detailed in the Data S7. The prognostic performance of the habitat pathomics‐based, clinical, and combined models was quantitatively evaluated using the concordance index (C‐index) and Time‐Dependent Curve. In addition, we distinguished patients based on the cutoff value of the best model and used the KM method to plot survival curves, and patients were further distinguished based on IDH status to investigate the discriminative ability of the model.

### 2.9 Statistical Analysis

The Shapiro–Wilk test was used to assess the normality of clinical characteristics. *T*‐tests were applied to continuous variables that were normally distributed, and the Mann–Whitney *U* test was employed for those that were not. Statistical significance for categorical variables was determined using the Chi‐square (*χ* ^2^) tests. Detailed information on patient characteristics is available in Table 1. The machine learning model was developed, and statistical analyses were performed using Python (version 3.7.12), Onekey (version 3.3.5), and scikit‐learn (version 1.0.2), with the training process aided by an NVIDIA 4090 GPU, employing MONAI (version 0.8.1) and PyTorch (version 1.8.1) frameworks.

**TABLE 1:** Baseline clinical characteristics of patients.

Characteristics	The entire cohort	The train cohort	The test cohort	*p*
	Number = 72	Number = 52	Number = 20	
Age	55.111 ± 12.209	55.577 ± 11.978	53.900 ± 13.030	0.605
Height	165.222 ± 7.947	164.673 ± 8.279	166.650 ± 7.006	0.371
Weight	65.054 ± 9.925	64.200 ± 9.063	67.275 ± 11.850	0.242
BMI	23.789 ± 3.025	23.681 ± 2.934	24.069 ± 3.313	0.629
Tumor volume	47.352 ± 38.582	40.777 ± 30.755	64.446 ± 50.968	0.051
Tumor area	1.944 ± 1.086	1.923 ± 1.100	2.000 ± 1.076	0.718
Sex				
Male	37 (51.389)	26 (50.000)	11 (55.000)	0.907
Female	35 (48.611)	26 (50.000)	9 (45.000)	
Chronic				
No	40 (55.556)	30 (57.692)	10 (50.000)	0.746
Yes	32 (44.444)	22 (42.308)	10 (50.000)	
Family disease				
No	71 (98.611)	51 (98.077)	20 (100.000)	1.0
Yes	1 (1.389)	1 (1.923)	Null	
Integrated diagnostic level				
3	22 (30.556)	12 (23.077)	10 (50.000)	0.053
4	50 (69.444)	40 (76.923)	10 (50.000)	
Pathological type				
Glioblastoma	48 (66.667)	39 (75.000)	9 (45.000)	0.046
Astrocytoma	10 (13.889)	5 (9.615)	5 (25.000)	
Oligodendroglioma	9 (12.500)	4 (7.692)	5 (25.000)	
Other	5 (6.944)	4 (7.692)	1 (5.000)	
Multifocal				
No	64 (88.889)	44 (84.615)	20 (100.000)	0.149
Yes	8 (11.111)	8 (15.385)	Null	
Tumor location				
Left	32 (44.444)	21 (40.385)	11 (55.000)	0.356
Right	37 (51.389)	28 (53.846)	9 (45.000)	
Bilateral	3 (4.167)	3 (5.769)	Null	
Beyond midline				
No	42 (58.333)	28 (53.846)	14 (70.000)	0.328
Yes	30 (41.667)	24 (46.154)	6 (30.000)	
IDH				
No	43 (59.722)	33 (63.462)	10 (50.000)	0.438
Yes	29 (40.278)	19 (36.538)	10 (50.000)	
Necrosis				
No	24 (33.333)	14 (26.923)	10 (50.000)	0.114
Yes	48 (66.667)	38 (73.077)	10 (50.000)	

## 3 Result

### 3.1 Baseline Patient Characteristics

The Radiation Therapy Center of the First Affiliated Hospital of Nanjing Medical University enrolled 234 patients with high‐grade gliomas for the first time from March 2015 to June 2023. After excluding 115 patients with missing histological slices or MRI image sequences, 40 patients with incomplete postoperative follow‐up data, and 7 patients with blurred MRI or slice image data, a final cohort of 72 patients (40 males and 32 females) was included in the analysis. Baseline characteristics such as age, gender, body mass index (BMI), multifocality, and tumor volume were assessed. The results of the intergroup comparison (*p* > 0.05) indicated that there were no significant differences between the two groups. The clinical data of this study are presented in Table 1.

### 3.2 Habitat Signature Generation

In our analysis, we tested the number of clusters ranging from 2 to 10 to evaluate their efficiency. We observed an initial increase in the Calinski–Harabasz, Silhouette, and Davies–Bouldin scores, followed by a decrease as the number of clusters increased. Based on these evaluations, the most favorable outcome was achieved with three cluster centers, indicating that three partitions are optimal for our habitat radiomics analysis.

**FIGURE 3:**
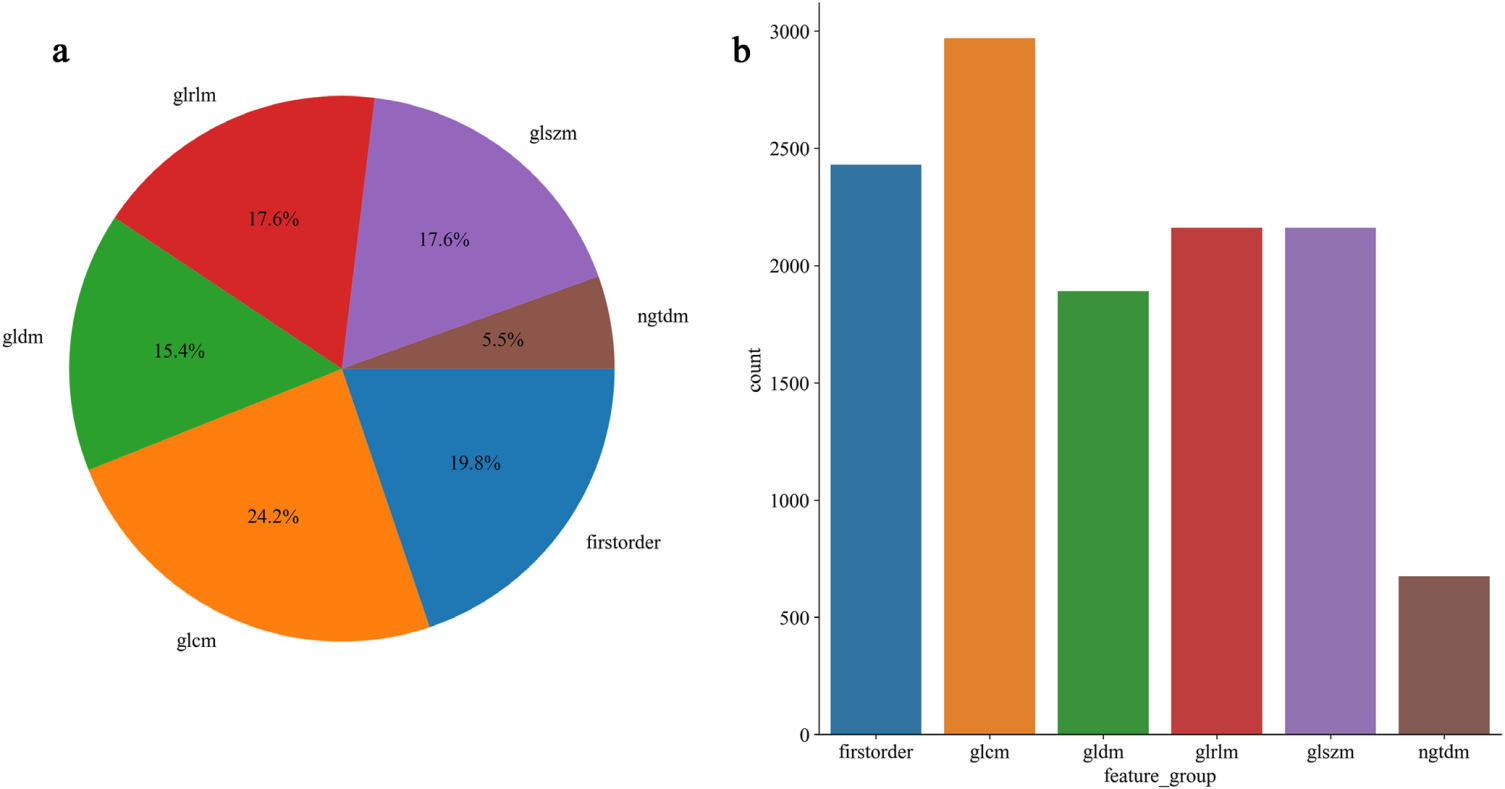
The six types of features extracted from the cluster regions, namely firstorder, glcm, gldm, glrlm, glszm, and ngtdm, along with their respective proportions.

Subsequently, in the clustering region, we extracted a total of 1834 distinct handcrafted habitat radiomics features, which were categorized into shape, first‐order, and texture features. This comprehensive feature extraction process utilized tools developed within the Pyradiomics framework. For more information about this tool, please visit http://pyradiomics.readthedocs.io. The extracted features are detailed in Figure 3.

Finally, we employed the Lasso method for feature selection, specifically using a LASSO logistic regression model. This approach helped us identify non‐zero coefficients, which are crucial for developing the Rad‐score.

### 3.3 Pathomics Signature Generation

Results of the AUC score analysis reveal that DenseNet121 achieved the top test performance, recording an AUC of 0.682 within a confidence interval of 0.6761–0.6878. In contrast, ResNet101 and Inception V3 had AUCs of 0.639 and 0.612, respectively, in the test cohort. DenseNet121 also exhibited relatively higher sensitivity of 0.771 and a negative predictive value (NPV) of 0.882, although it had lower specificity (0.530) and a positive predictive value (PPV) of 0.336.

Given its superior AUC and reasonable balance between sensitivity and NPV, DenseNet121 was selected as the model for the multiple instance learning model to characterize features within our study. This choice is predicated on the need for a robust predictive model that optimally balances generalization with precision in real‐world scenarios. The integration of DenseNet121 into our multi‐instance learning framework is expected to enhance the model's ability to accurately profile histopathological signatures. The corresponding information can be found in Table 2.

**TABLE 2:** WSI level accuracy and AUC of each model.

Model name	Acc	AUC (95% CI)	Sensitivity	Specificity	PPV	NPV	Cohort
densenet121	0.954	0.992 (0.9915–0.9923)	0.952	0.957	0.954	0.955	Train
densenet121	0.586	0.682 (0.6761–0.6878)	0.771	0.530	0.336	0.882	Test
resnet101	0.974	0.997 (0.9968–0.9972)	0.974	0.974	0.972	0.975	Train
resnet101	0.502	0.639 (0.6332–0.6451)	0.843	0.397	0.302	0.891	Test
inception_v3	0.978	0.998 (0.9978–0.9981)	0.981	0.976	0.974	0.982	Train
inception_v3	0.542	0.612 (0.6057–0.6183)	0.681	0.499	0.296	0.835	Test

By combining patch‐level predictions, probability histograms, and TF‐IDF features, we ultimately generated 5 pathological features through Pearson correlation analysis, univariate Cox regression, and LASSO‐Cox regression.

### 3.4 Visualization

#### 3.4.1 Intratumoral Heterogeneity Visualization

**FIGURE 4:**
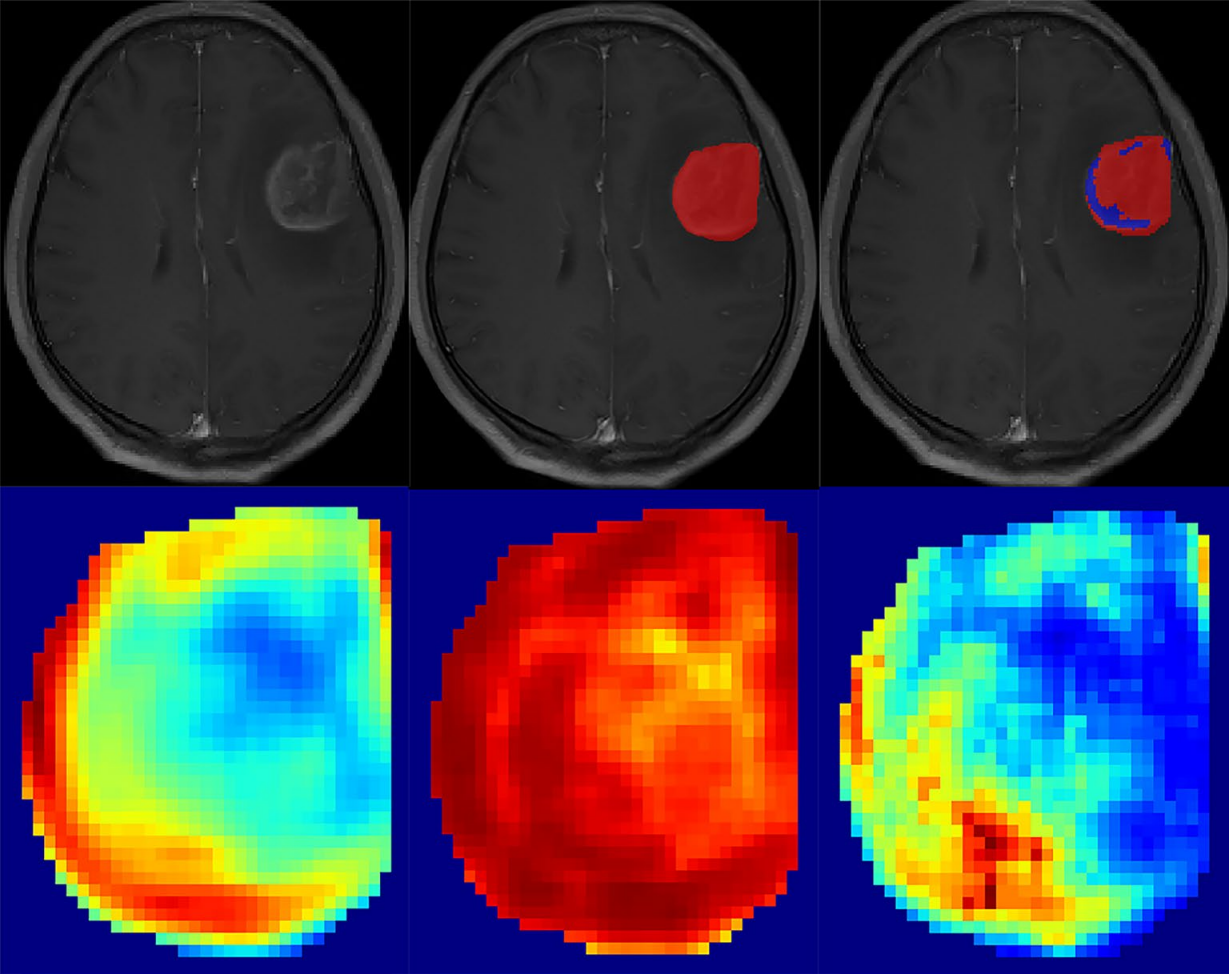
Intratumor Heterogeneity Regions.

To visually illustrate the concept, we have generated visual representations of typical clustering outcomes. These illustrations depict a specific case of a patient diagnosed with grade 4 glioblastoma located in the left temporal lobe and exhibiting a negative IDH mutation. In these images, the tumor is clearly visible, with a distinct central necrotic area surrounded by a region displaying discernible characteristics. The information can be found in Figure 4.

#### 3.4.2 Gradient‐Weighted Class Activation Mapping Visualization

**FIGURE 5:**
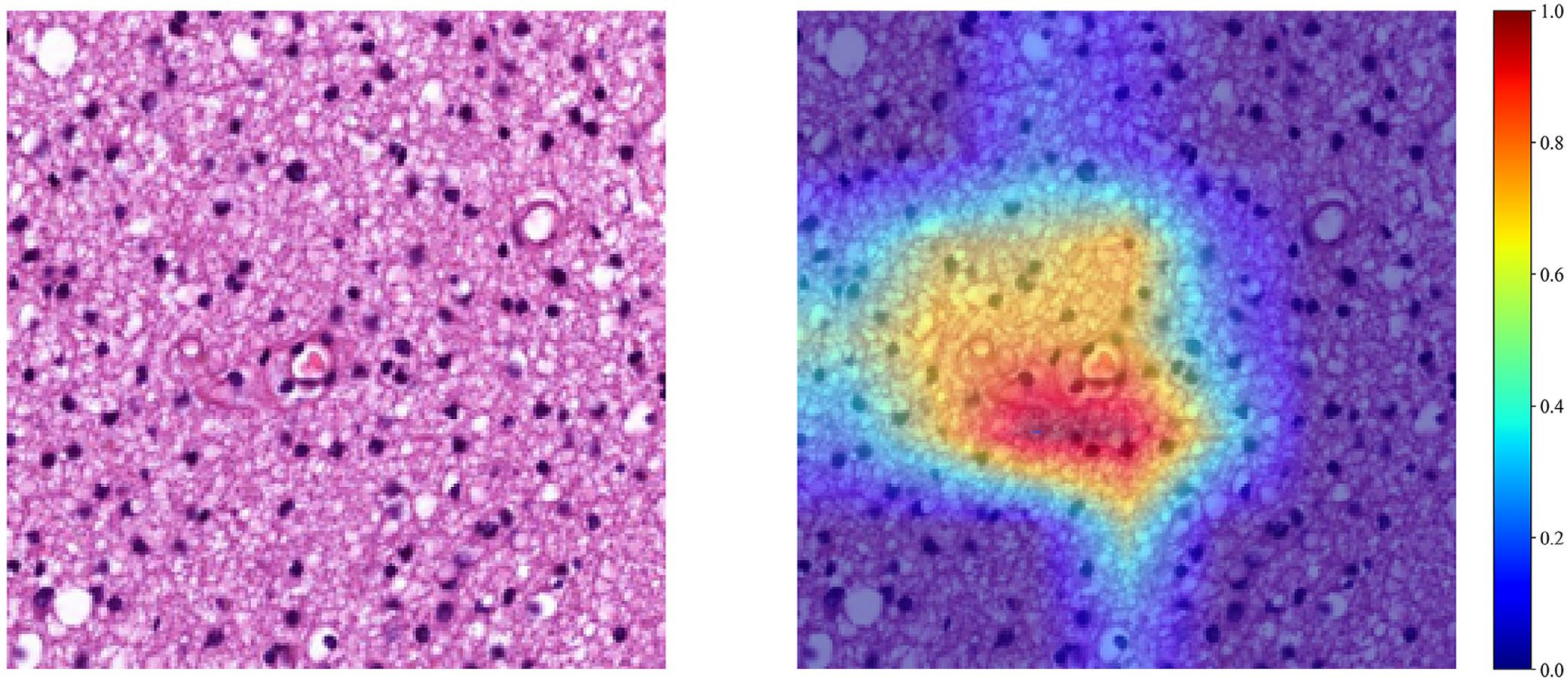
Grad‐CAM of patch.

We employed the Gradient‐weighted Class Activation Mapping (Grad‐CAM) method to visualize and assess the recognition capabilities of deep learning models on different samples, emphasizing the activations in the last convolutional layer that are pertinent to predicting cancer types. This helps in identifying image regions that significantly impact the model's decision‐making, offering insights into its interpretability, and the related information can be found in Figure 5. We also provide the prediction visualizations for some samples, and the related information can be found in Figure S2.

#### 3.4.3 Seaborn Cluster Heatmap of Habitat Radiomics Features and Pathological Features

**FIGURE 6:**
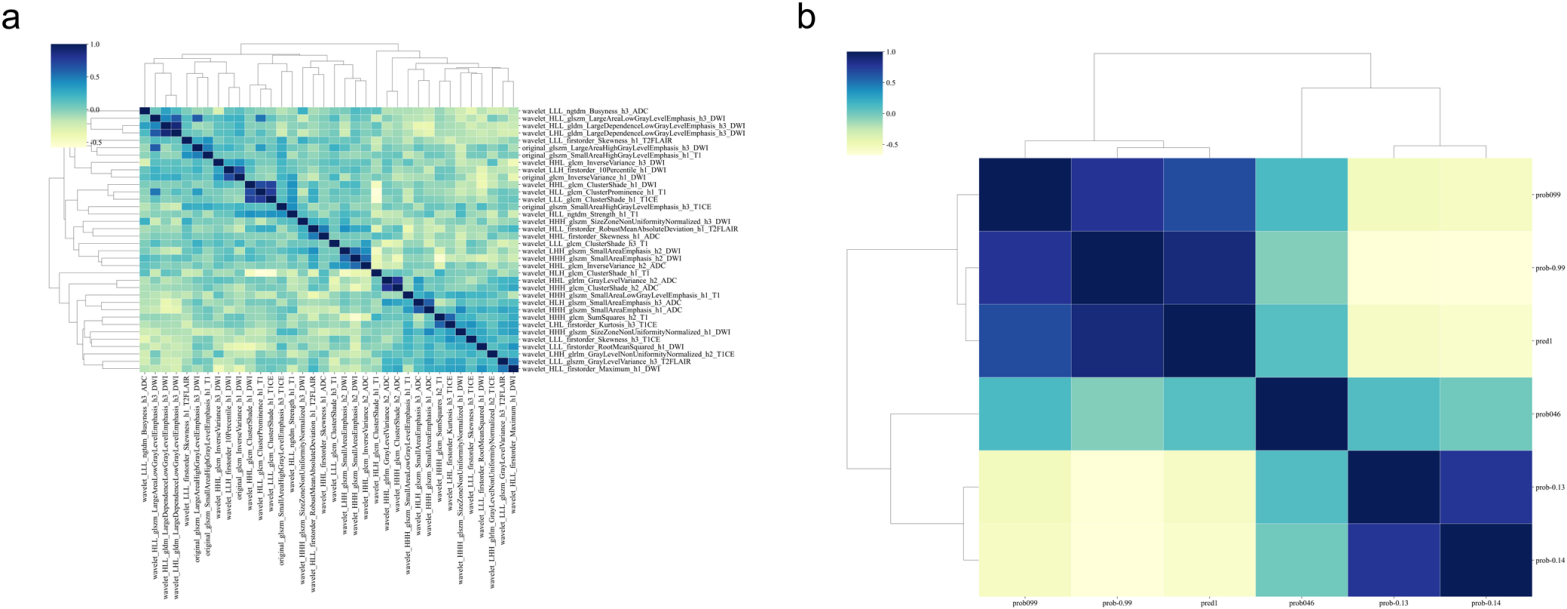
(a) Seaborn cluster heatmap of habitat radiomics features; (b) Seaborn cluster heatmap of pathological features.

We adopted the Seaborn Clustered Heatmap method to visualize and evaluate the degree of correlation between different features. This helps to intuitively display the structure of the data, enabling researchers to quickly identify similarities and differences among features. For related content, please refer to Figure 6.

### 3.5 Model Construction and Predictive Performance

#### 3.5.1 Model Construction and Signature Comparison

For the train cohort, the Combined model showed an improvement in C‐index for progression‐free survival (PFS), with values of 0.883. This represents an enhancement over the individual contributions from the Clinical, Pathomics‐based, and Habitat models, which posted scores of 0.719, 0.849, and 0.867. Similarly, in the test cohort, the Combined model outperformed the individual models with the highest scores of 0.840, compared with the next highest scores of 0.825 (Habitat). The C‐index values for different models have been detailed in Table 3 for reference.

**TABLE 3:** C‐index in prediction PFS.

Model	Train cohort		Test cohort	
	C‐index	*p* ^a^	C‐index	*p* ^a^
Habitat model	0.867	< 0.001	0.825	< 0.001
Pathomics‐based model	0.849	< 0.05	0.740	< 0.05
Clinical model	0.719	0.0006	0.730	0.0651
Combined model	0.883	< 0.05	0.840	< 0.05

^a^ The *p*‐value here is the *p*‐value for the KM method of each model.

#### 3.5.2 Time‐Dependent ROC Analysis

**FIGURE 7:**
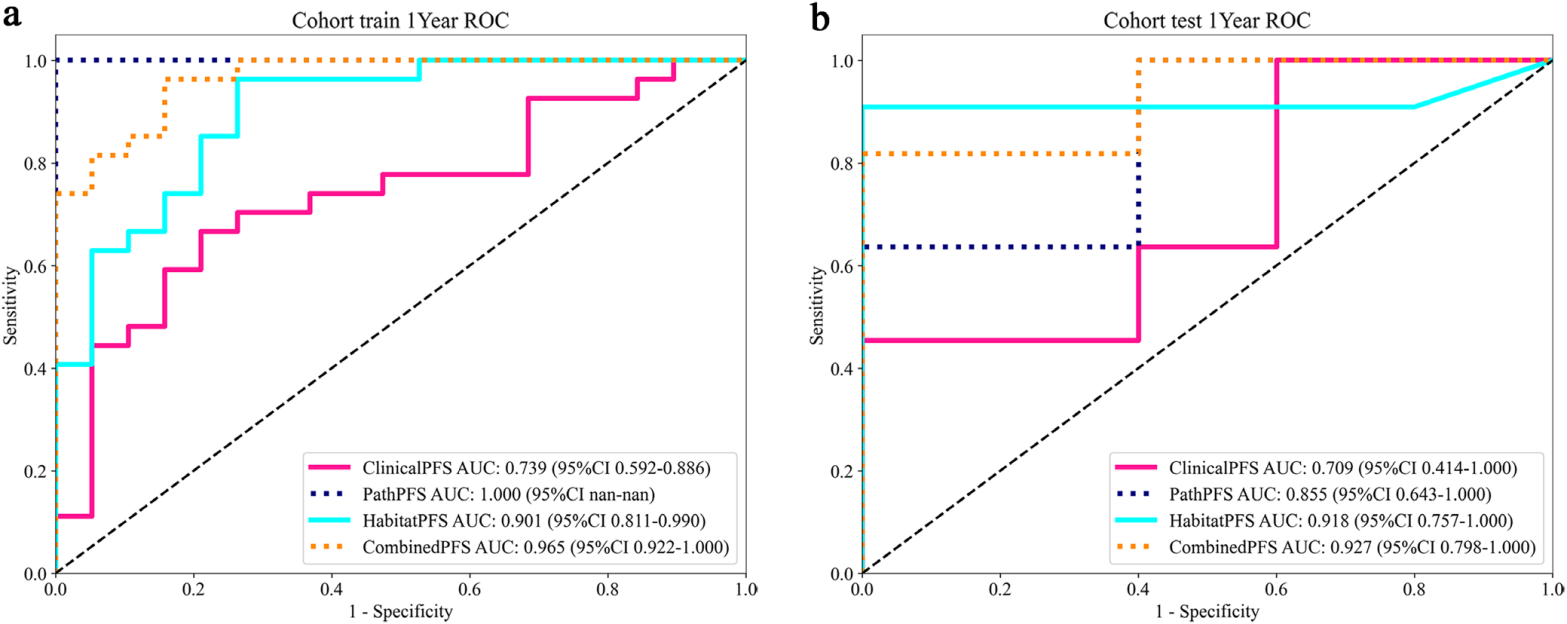
The ROC curves of each model in the train and test cohorts (a, b).

In the train cohort, the pathomics‐based model achieved the highest AUC of 1.000, outperforming the combined model (AUC = 0.965), habitat model (AUC = 0.901), and the clinical model (AUC = 0.739). It should be noted that the AUC of 1.0 corresponds to a time‐dependent ROC analysis at a specific follow‐up time point with a limited number of cases. This can sometimes lead to an inflated AUC due to the small sample size at that time point, rather than indicating perfect model performance. Within the test cohort, the combined model achieved the highest AUC of 0.927, with the habitat model following at 0.918, pathomics‐based model at 0.855, and clinical model at 0.709. These results indicate that the combined model exhibits superior AUC scores, particularly in the test cohort, indicating its robust performance and potential predictive ability. This highlights its robustness and potential applicability in a clinical setting. The relevant ROC curve is shown in Figure 7.

#### 3.5.3 Risk Stratification and IDH Status Distinguish Prognosis

**FIGURE 8:**
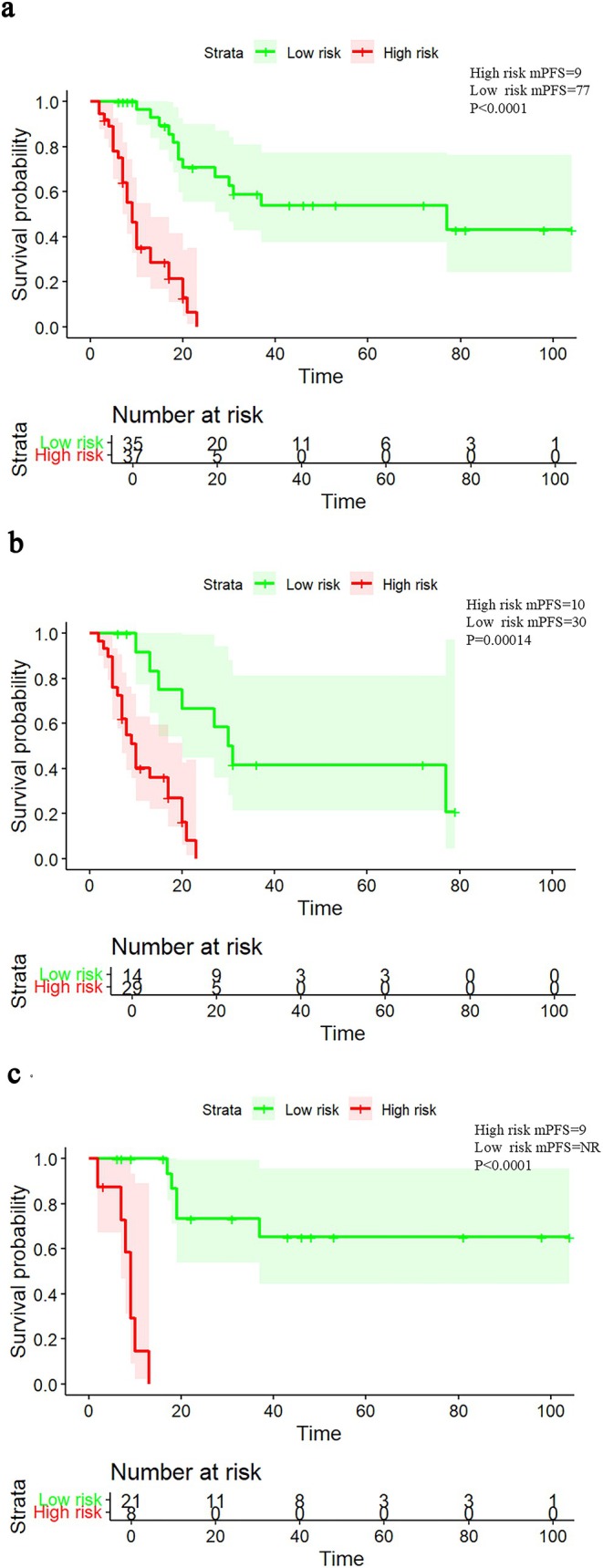
(a) Progression Free Survival curve of all patients, (b) Progression Free Survival curve of patients with IDH (−), (c) Progression Free Survival curve of patients with IDH (+). (NR, not reach).

In our study, the combined model demonstrated superior performance in both the train and test cohorts compared to other models, thereby establishing it as the preferred model for further analysis. Based on the clinical progression of patients, we categorized them into two groups and constructed survival curves (*p* < 0.0001) according to whether they progressed within 1 year (Figure S3). The median progression‐free survival (PFS) and median overall survival (OS) in the group without progression within 1 year were significantly longer than those in the group with progression within 1 year. Specifically, the median PFS was 31 months for the non‐progression group compared to 8 months for the progression group, while the median OS was 56 months for the non‐progression group compared to 15 months for the progression group. Subsequently, we utilized the combined model to divide patients into high‐risk and low‐risk groups based on the survival curves (*p* < 0.0001). The median PFS in the low‐risk group was also significantly longer than that in the high‐risk group, at 77 and 9 months, respectively. In the IDH (−) population (*p* = 0.00014), the median PFS for the high‐risk group was 10 months, while the low‐risk group exhibited a more favorable prognosis with a median PFS of 30 months. A similar trend was observed in the IDH (+) population (*p* < 0.0001), where the median PFS for the high‐risk group was 9 months. These findings indicate that the combined model has a significant stratification effect and can effectively distinguish between high‐risk and low‐risk patients. The relevant survival curves can be found in Figure 8.

## 4 Discussion

The predictive model based on habitat combined with pathomics established in this study has important clinical application value for the progression risk prediction of high‐grade gliomas. In this study, we categorized patients based on whether they progressed within 1 year, and there was a significant difference in the KM survival curves between the two groups. We constructed the habitat model, pathomics‐based model, and clinical model using multiple MRI sequences, WSI, and clinical data, respectively, and then integrated these three models to build a combined model. The results showed that the combined model demonstrated good prognostic value in both the train cohort (*C*‐index = 0.883, AUC = 0.965) and the test cohort (*C*‐index = 0.840, AUC = 0.927) and was comprehensively evaluated as the optimal model. Furthermore, we performed patient stratification based on the optimal model, combined with IDH information, which further improved the accuracy of prediction and supplemented the prognostic information for patients.

Currently, there are relevant studies exploring the application of radiopathomics in oncology, and this approach has been proven to provide complementary information about tumor biology [25, 28]. Radiomics is an important field in oncology that utilizes quantitative features extracted from medical images and converts them into precise and comprehensive lesion assessment indicators, thereby offering clinically relevant diagnostic and prognostic insights. It can reveal tumor heterogeneity at the macro level to improve predictive models [11, 36]. However, predictions based solely on radiomic features may overlook crucial biological and pathological information related to tumor prognosis [37]. To address this limitation, we introduced pathological features in this study. Pathomics is a novel tool capable of comprehensively extracting features, with the potential to enhance tumor outcome prediction. Compared with radiomics, which focuses on the macro level, pathomics can reveal changes in tumor cell density, distribution, and morphology, supplementing information at the micro level of tumors, thereby improving model predictive performance [27, 29]. However, there is currently limited research on the application of habitat radiomics analysis combined with pathomics in gliomas. The study conducted by Saima Rathore and colleagues included a population encompassing both low‐grade and high‐grade gliomas [38]. In contrast to their study, our selected population is composed of high‐grade gliomas, which hold greater clinical relevance. This approach eliminates the heterogeneity associated with different grades of gliomas, thereby simplifying prognostic analyses. Moreover, we incorporated more MRI sequences, such as DWI and ADC sequences, both of which provide crucial insights into tissue microstructure, cellular architecture, and the integrity of white matter. In terms of radiomics, we adopted habitat analysis, a method based on Darwinian evolutionary dynamics that combines information about tumor cells and their microenvironment to better reveal the spatial heterogeneity of tumors, which is beneficial for better characterization of habitat radiomics features. Therefore, the AUC and C‐index of our study are both higher than those of Saima Rathore and others. The addition of multiple sequences has improved the predictive performance of the model, contributing to a more comprehensive prediction of prognosis. Regarding pathomics, we employed deep learning and multi‐instance learning techniques. These methodologies improve generalization capability, enhance model performance, and increase the accuracy of outcome predictions, thereby playing an essential role in prognostic assessment. Additionally, the performance of the combined model in our study is superior to that of other individual models, which is consistent with the results of studies on breast cancer, gastric cancer, prostate cancer, and other cancers [39, 40, 41]. We also examined the relationship between our model‐based risk stratification and IDH status, an important molecular marker in glioma. Patients with IDH wild‐type and high‐risk status had short progression‐free survival (PFS), which is consistent with the previous research findings of Wu H and Choate KA et al. [42, 43]. Additionally, in the population with IDH mutations, we further screened out a subgroup with poor prognosis based on this model, with a median PFS of 10 months in this subgroup. To avoid rapid progression, these patients should receive intensified treatment to improve their prognostic survival time and quality of life. This highlights the valuable insights provided by our model‐based grouping approach regarding the prognostic implications associated with IDH status in high‐grade gliomas.

In clinical practice, this combined model can serve as an auxiliary tool for risk stratification in patients with high‐grade glioma, thereby providing a quantitative basis for clinical decision‐making. Based on the risk stratification output by the model, clinicians can formulate more individualized therapeutic strategies. This study has demonstrated that the model can effectively identify the patient population at high risk of progression. For this specific cohort, clinicians may prioritize the following interventions when making treatment decisions: (1) Whether to adjust the radiation dose and irradiation range during the concurrent radiotherapy combined with TMZ. (2) Whether to consider combining electric field therapy or anti‐angiogenic therapy during the concurrent radiotherapy phase and the 6‐cycle oral TMZ phase [6, 44]. (3) Consider extending the duration of maintenance therapy to achieve a better prognosis. (4) Attempt to administer medication preoperatively to obtain better surgical conditions and improve the quality of life after treatment. (5) For patients with a poorer prognosis, more frequent follow‐ups can help detect changes in their condition earlier, allowing for timely treatment. For low‐risk patients, the following measures can be considered: (1) Consider appropriately reducing the radiotherapy dose to lower the side effects experienced by the patient during radiotherapy. (2) Appropriate extension of patient follow‐up intervals can reduce their economic burden. Future clinical applications will require prospective studies, such as non‐inferiority clinical trials, to further validate the feasibility and efficacy of the proposed interventions. At the clinical decision‐making level, this model can facilitate evidence‐based and informed communication between physicians and patients, enabling the co‐creation of personalized treatment plans. At the healthcare resource management level, the model's accurate prognostic prediction capabilities can aid healthcare institutions in optimizing resource allocation, particularly in radiotherapy planning and subsequent care management. Through the precise identification and focused management of high‐risk patients, the intensive utilization of medical resources can be achieved, ultimately enhancing the overall efficiency and quality of healthcare services.

The limitations of this study warrant attention in future research. First, this was a single‐center retrospective study with a limited sample size, which restricts the model's internal and external generalizability. The limited sample size stemmed from three main factors: first, the inherent limitations of interdisciplinary collaboration and pathological workflow management prevented the acquisition of pathological slides for some patients; second, the initial diagnostic MRI scans for some patients were performed at external institutions, and the heterogeneity of their scanning protocols precluded the standardization and integration of the corresponding data. Second, the study did not incorporate other omics data (such as functional imaging and genomics), leading to suboptimal biological interpretability, and an attention mechanism was not included. Finally, this study only predicted progression‐free survival (PFS) without including the crucial metric of overall survival (OS). To further enhance the model's predictive accuracy and generalizability, our future work will focus on: (1) continuously expanding the sample size by conducting prospective multicenter studies to achieve more robust external validation; and (2) strengthening interdisciplinary collaboration to systematically integrate multi‐omics data and introducing an attention mechanism to enhance the model's predictive performance and uncover deeper biological correlations.

This study established a model for predicting the risk of progression in high‐grade gliomas based on habitat radiomics and pathomics. The combined model, which integrates clinical data, pathological features, and habitat radiomics features, outperforms other models in terms of predictive performance. Furthermore, the model is capable of stratifying patient survival based on IDH status, which further enhances its predictive power. This study hopes to offer new perspectives and references for the personalized treatment and prognostic assessment of high‐grade gliomas.

## Author Contributions

Yuchen Zhu, Yuxi Gong, and Weilin Xu contributed equally to this article and shared first authorship. Lei Qiu, Kexin Shi, and Mengxing Wu collected clinical data. Yinjiao Fei and Jinling Yuan collected WSI images. Jinyan Luo and Yurong Li completed the data sorting and induction. Yuxi Gong completed the selection of ROI regions. Yuchen Zhu, Gefei Jiang, and Xingjian Sun analyzed the data and wrote the manuscript. Weilin Xu validated the ROI. Yuandong Cao, Minhong Pan, and Shu Zhou provided study supervision and article revision. All authors contributed to the article and approved the submitted version.

## Funding

The authors have nothing to report.

## Ethics Statement

This study was performed in line with the principles of the Declaration of Helsinki. Approval was granted by the Ethics Committee of Nanjing Medical University First Affiliated Hospital (No. 2024‐SR‐913). Informed consent was waived because it was a retrospective study.

## Conflicts of Interest

The authors declare no conflicts of interest.

## Supporting information


**Data S1:** acn370304‐sup‐0001‐DataS1.docx.

## Data Availability

The original data of this study involves patient privacy and cannot be shared on a database. The research data can be shared by the primary contact person upon request. This paper does not report the original code. Any additional information required to reanalyze the data reported in this paper can be obtained from the primary contact person upon request.
