# Supplementary material for: Development of a Prediction Model for Progression Risk in High‐Grade Gliomas Based on Habitat Radiomics and Pathomics
Source: Ann Clin Transl Neurol. 2026 Jan 4;13(6):1141–53. doi: 10.1002/acn3.70304 (PMC13251424; doi:10.1002/acn3.70304)
Supplement: Supplementary file 1 — Data S1: acn370304‐sup‐0001‐DataS1.docx. [file ACN3-13-1141-s001.docx]

**Development of a Prediction Model for Progression Risk in High-Grade Gliomas Based on Habitat Radiomics and Pathomics**

**Content：**

**Supplementary Material A1.MRI scan protocols**

**Supplementary Material A2. The Generation Process of Feature Vector**

**Supplementary Material A3. Habitat Generation Process**

**Supplementary Material A4. Feature Extraction and Selection**

**Supplementary Material A5. Slice Level Model Training**

**Supplementary Material A6. Multi Instance Learning for WSI Fusion**

**Supplementary Material A7. model construction method**

**Supplementary Fig.1 Examples of relevant ROI delineation**

**Supplementary Fig.2 Visualize predictions**

**Supplementary Fig.3 Prognosis of progression groups within one year**

**Supplementary Eq. (A.1). K-means algorithm**

**Supplementary Material A1. MRI scan protocols**

**Images were acquired in the routine clinical work-up on a 3.0-T MRI system (Magnetom Verio Tim; Siemens, Erlangen, Germany) with a 12-channel head matrix coil. The conventional MRI protocols consisted of the following sequences: T1W, T1CE, , T2FLAIR and DWI. The MRI scan protocols and main parameters are follows:**

| **Sequence** | **FOV** | **TR** | **TE** | **Slice thickness** | **Voxel size** |
| --- | --- | --- | --- | --- | --- |
|  | **(mm)** | **(ms)** | **(ms)** | **(mm)** | **(mm)** |
| **T_1_ W** | **230** | **1800** | **9** | **5** | **0.7×0.7×5.0** |
| **T2FLAIR** | **230** | **8000** | **97** | **5** | **0.4×0.4×5.0** |
| **DWI** | **220** | **4480** | **69** | **5** | **1.1×1.1×5.0** |
| **T1CE** | **230** | **400** | **2.48** | **5** | **0.7×0.7×5.0** |

**DWI was acquired in three orthogonal directions, and the images are combined into a trace image. DWI was obtained using the following parameters: 4480 ms TR, 69 ms TE, average=2, 5 mm section thickness, 1 mm intersection gap, 220×220 mm FOV; diffusion gradient encoding, b = 0, and 1000 s/mm^2^. Processing of the ADC map was generated automatically by the MRI system. *TE, echo time; TR, repetition time; FLAIR, fluid-attenuated inversion recovery; DWI, diffusion-weighted imaging; CE, contrast-enhanced.***

**Supplementary Material A2. The Generation Process of Feature Vector**

**Comprehensive Radiomic Feature Extraction: This process involved extracting detailed local features from each voxel in the dataset using a** $\boldsymbol{3}\boldsymbol{\times}\boldsymbol{3}\boldsymbol{\times}\boldsymbol{3}$ **moving window. These features encompass a variety of measurements and attributes, including intensity, texture, and other statistical properties, which are crucial for understanding the intricate details of the dataset. Such detailed insights enable more precise modeling and analysis.**

**In this study, 19 radiomic features were extracted from each voxel, offering a multidimensional characterization of each subregion. These features included a range of shape descriptors, textural features, and first-order statistical attributes. The specific features extracted were: firstorder_Entropy, firstorder_MeanAbsoluteDeviation, firstorder_Median, glcm_DifferenceAverage, glcm_DifferenceEntropy, glcm_DifferenceVariance, glcm_Imc1, glcm_Imc2, glcm_InverseVariance, glcm_JointEnergy, glcm_JointEntropy, glcm_SumEntropy, glrlm_LongRunEmphasis, glrlm_RunEntropy, glrlm_RunVariance, glszm_SizeZoneNonUniformityNormalized, glszm_SmallAreaHighGrayLevelEmphasis, ngtdm_Contrast, and ngtdm_Strength**.

**Supplementary Material A3. Habitat Generation Process**

Habitat Region Synthesis: Following the clustering analysis, subregions with identical cluster IDs were amalgamated. This synthesis resulted in the formation of comprehensive habitat regions, each representing a unique microenvironmental characteristic within the tumor.

This extensive process underscores the complexity and thoroughness of our approach in segmenting and analyzing tumor habitat regions. Our methodology not only enhances the depth of our tumor microenvironment study but also contributes to the broader field of medical imaging and radiomics.

**Supplementary Material A4. Feature Extraction and Selection**

Feature Extraction

In our study, we focused on T1CE data to extract features from generated tumor heterogeneity regions, organizing handcrafted features into three primary categories for effective extraction: Geometry (capturing the tumor's shape), Intensity (analyzing the distribution of voxel intensities), and Texture (examining intensity patterns and spatial distribution using techniques like GLCM). Utilizing Pyradiomics version 3.0.1, we adhered to the Imaging Biomarker Standardization Initiative guidelines for feature extraction. This process was performed independently across specified regions within each imaging modality, accommodating the variability in the number and distribution of subregions. For areas lacking sufficient voxel counts, we applied a k-nearest neighbors strategy to address missing data.

Feature Selection

Correlation Analysis: We employed Pearson's correlation coefficient to identify highly repeatable features. Features with a correlation coefficient exceeding 0.9 led to the retention of only one feature to prevent redundancy. This was complemented by a recursive feature elimination strategy, where the most redundant feature was systematically excluded in each iteration.

Univariable Cox Regression: To refine the extensive feature set, we conducted univariable Cox regression analysis. Features were ranked based on their p-values. This method proved more efficacious in enhancing predictive performance than merely selecting features with p-values below 0.05, as demonstrated in our experiments.

Lasso-Cox Regression: This study employed LASSO (Least Absolute Shrinkage and Selection Operator) Cox regression analysis to construct the final feature set for the signature. This method eliminates irrelevant features by shrinking their coefficients to zero, a process that is contingent on the regularization parameter λ. We determined the optimal λ value on the training set using 10-fold cross-validation, by selecting the value that minimized the mean standard error.

**Supplementary Material A5. Slice Level Model Training**

**Model Structure**

**CNN Characteristics: Convolutional Neural Networks (CNNs) are particularly effective for image processing due to their ability to capture hierarchical patterns and features through convolutional layers. These networks employ filters to perform convolution operations that extract spatial hierarchies of features, making them adept at handling image data.**

**Data Augmentation: To ensure a uniform input distribution, Z-score normalization was applied to the RGB channels of images. Our model benefited from online data augmentation techniques such as random cropping and flipping, both horizontal and vertical, to increase the variability and robustness of the training data. For testing, normalization was the sole preprocessing step to maintain consistency.**

**Training: The use of transfer learning, with initialization from pre-trained ImageNet weights, leverages rich feature representations developed for broad visual recognition tasks, thus enhancing the model’s adaptability to our specific medical imaging context. The adoption of a cosine decay learning rate algorithm further aids in managing the learning rate throughout training to avoid local minima and stabilize convergence. Specifically, this approach adjusts the learning rate according to the formula:**

**η_t=η_min^i+1/2 (η_max^i-η_min^i )(1+cos(T_cur/T_i π))**

**The minimum and maximum learning rates are set to 0 and 0.01, respectively, with a cycle of 10 epochs. Training was optimized using Stochastic Gradient Descent (SGD), and softmax cross-entropy was employed for calculating loss, which enhances the discriminative training of deep networks for multi-class classification.**

**Training Parameters**

**The relevant parameters are: Batch_Size=32, epoch=10, lr=0.01. The method uses cosine-based decay, and the optimizer is SGD. We employed early stopping, stopping the training if the iteration count does not decrease after 32 iterations.**

**Supplementary Material A6. Multi Instance Learning for WSI Fusion**

Following the completion of the training phase for our deep learning model, we advanced to the stage of predicting labels and their corresponding probabilities for each individual patch extracted from whole slide images (WSI). These probabilities were not evaluated in isolation; instead, they were integrated using sophisticated classifiers to derive cohesive predictions at the WSI level. To effectively aggregate the probabilities associated with individual patches, we implemented two innovative machine learning methodologies designed to capture and utilize the complex data structure inherent in WSIs:

1. Patch Prediction: Each slice was analyzed using the deep learning model to derive probabilities and labels, denoted as $\boldsymbol{Patc}\boldsymbol{h}_{\boldsymbol{prob}}$ and $\boldsymbol{Patc}\boldsymbol{h}_{\boldsymbol{pred}}$, retained to two decimal places.
2. Multi Instance Learning Feature Aggregation:
   - Histogram Feature Aggregation:
     - Distinct numbers were treated as "bins" to count occurrences across types.
     - Frequencies of $\boldsymbol{Patc}\boldsymbol{h}_{\boldsymbol{prob}}$ and $\boldsymbol{Patc}\boldsymbol{h}_{\boldsymbol{pred}}$ in each bin were tallied and normalized using min-max normalization, resulting in $\boldsymbol{Hist}\boldsymbol{o}_{\boldsymbol{prob}}$ and $\boldsymbol{Hist}\boldsymbol{o}_{\boldsymbol{pred}}$.
   - Bag of Words (BoW) Feature Aggregation:
     - A dictionary was constructed from unique elements in $\boldsymbol{Patc}\boldsymbol{h}_{\boldsymbol{prob}}$ and $\boldsymbol{Patc}\boldsymbol{h}_{\boldsymbol{pred}}$.
     - Each slice was represented as a vector noting the frequency of each dictionary element, with a TF-IDF transformation applied to emphasize informative features.
     - This resulted in a BoW feature representation for each slice, encapsulating both the presence and significance of features.
3. Feature Early Fusion: We integrated $\boldsymbol{Hist}\boldsymbol{o}_{\boldsymbol{prob}}$, $\boldsymbol{Hist}\boldsymbol{o}_{\boldsymbol{pred}}$, $\boldsymbol{Bo}\boldsymbol{w}_{\boldsymbol{prob}}$, and $\boldsymbol{Bo}\boldsymbol{w}_{\boldsymbol{pred}}$ using a feature concatenation method ($\boldsymbol{\oplus}$), combining these into a single comprehensive feature vector:

$$\boldsymbol{featur}\boldsymbol{e}_{\boldsymbol{fusion}}\mathbf{=}\boldsymbol{Hist}\boldsymbol{o}_{\boldsymbol{prob}}\boldsymbol{\oplus}\boldsymbol{Hist}\boldsymbol{o}_{\boldsymbol{pred}}\boldsymbol{\oplus}\boldsymbol{Bo}\boldsymbol{w}_{\boldsymbol{prob}}\boldsymbol{\oplus}\boldsymbol{Bo}\boldsymbol{w}_{\boldsymbol{pred}}$$

**Supplementary Material A7. model construction method and Cross-validation of the habitat model**

**Model Construction**

**Habitat radiomics model: Following Lasso feature screening, Cox regression was employed to model the selected features and estimate the average expected survival time, resulting in the development of our habitat radiomics signature. It should be emphasized that the Habitat Radiomics Signature, derived from habitat radiomics results, provides a unique perspective on comprehending the intricacies of intratumoral heterogeneity.**

**Pathomics-Based Model:** **Following Lasso feature screening, Cox regression was employed to model the selected features and estimate the average expected survival time, resulting in the development of our Histopathological signature.**

Clinical model: We incorporated clinical characteristics into a Cox model and designated the anticipated survival time as the Clinical Signature.

Combined Model: To assess the effectiveness of a multi-omics strategy, the Clinical Signature, Habitat Radiomics Signature, and Histopathological Signature were combined using a Cox model to create a Combined Model.

**Supplementary Fig.1 Examples of relevant ROI delineation**

**
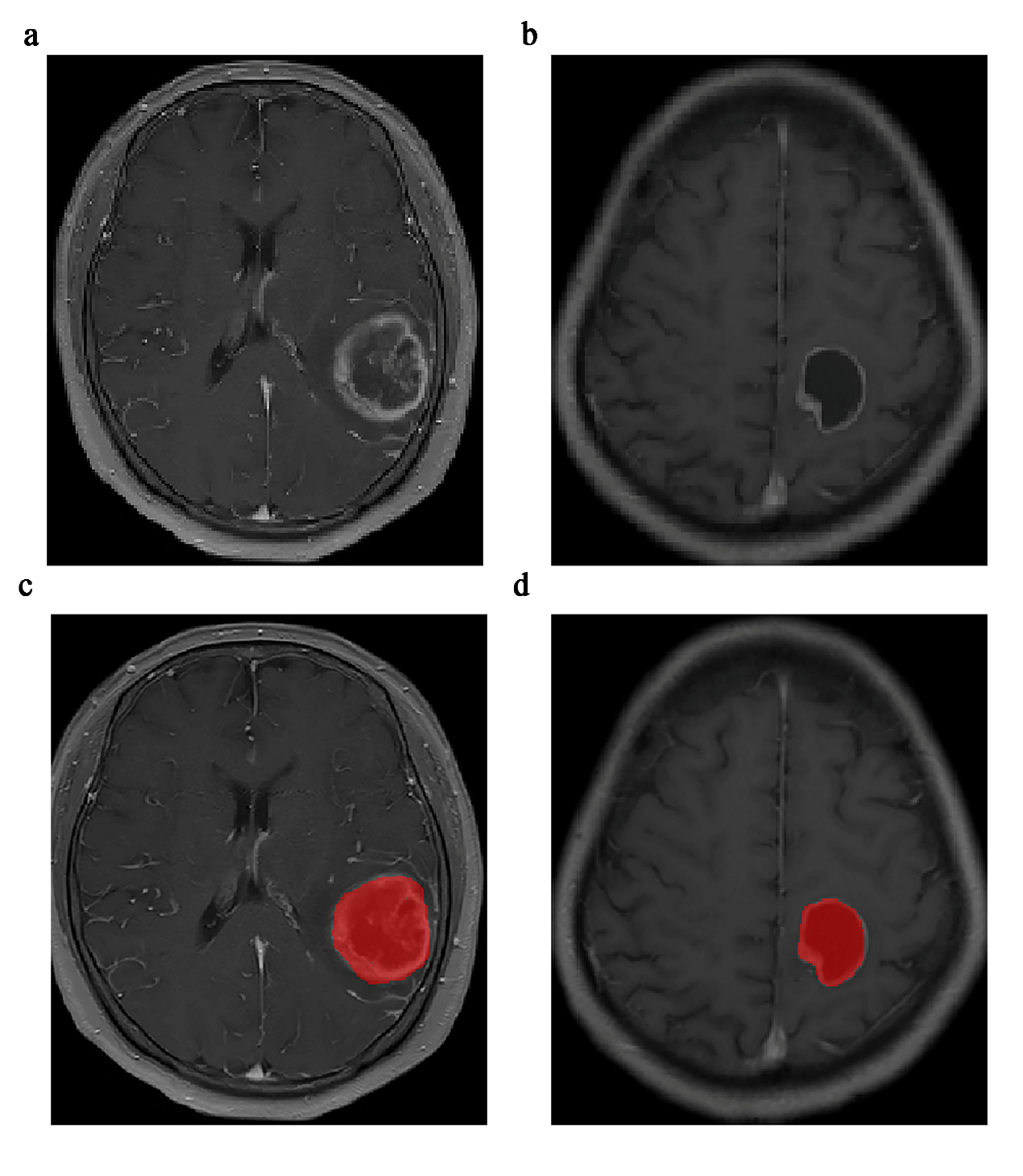
**

**Supplementary Fig.1 relevant ROI delineation of 1(a, c), 12(b, d) sample**

**Supplementary Fig.2 Visualize predictions**

This prediction result of prediction 25, 27 sample. It is evident that our pathological model exhibits a high level of accuracy in predicting [TaskSpec] tiles.

**
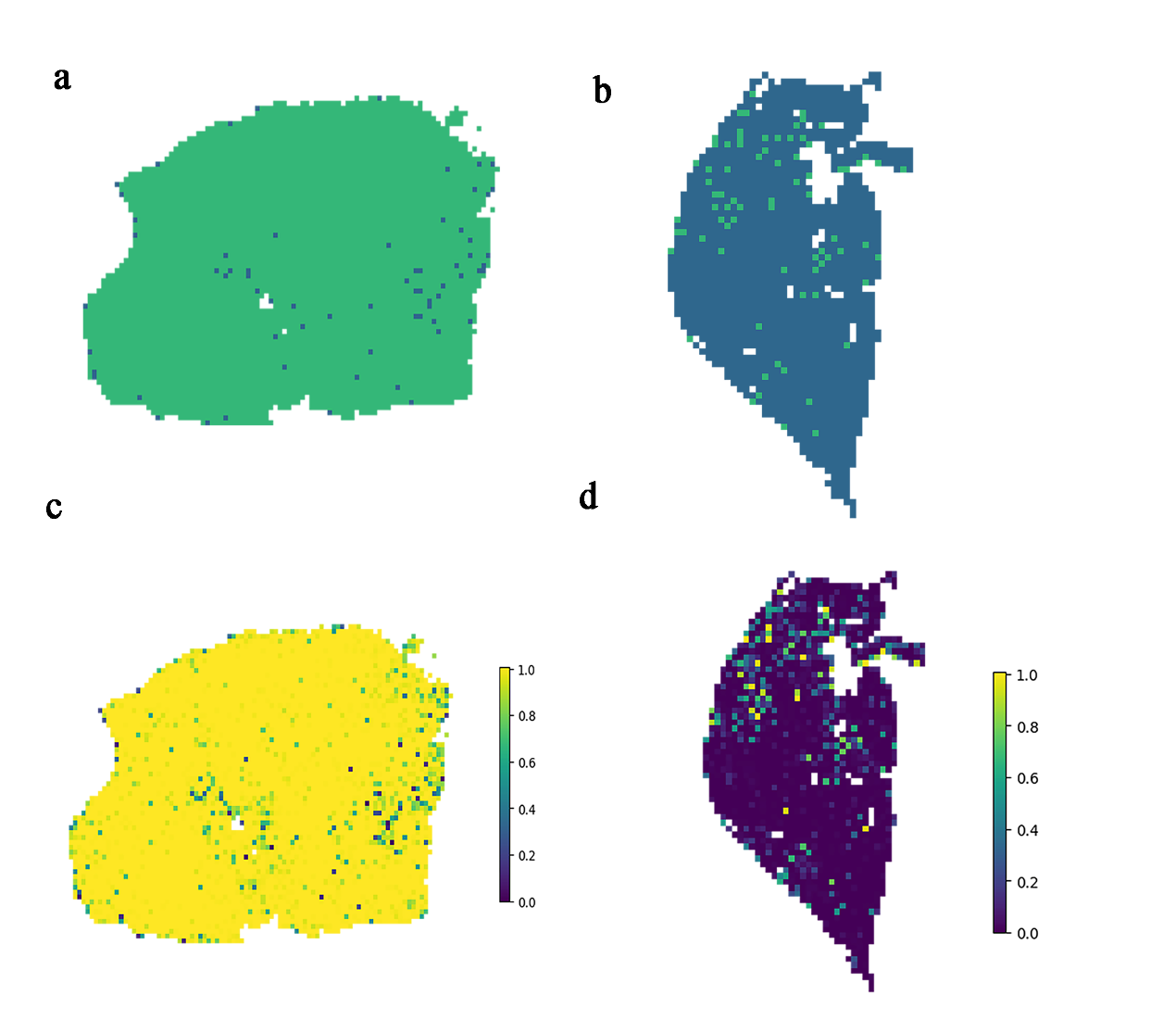
**

Supplementary Fig.2 Prediction (a, b) and probably (c, d) map of 25(a, c), 27(b, d) sample.

**
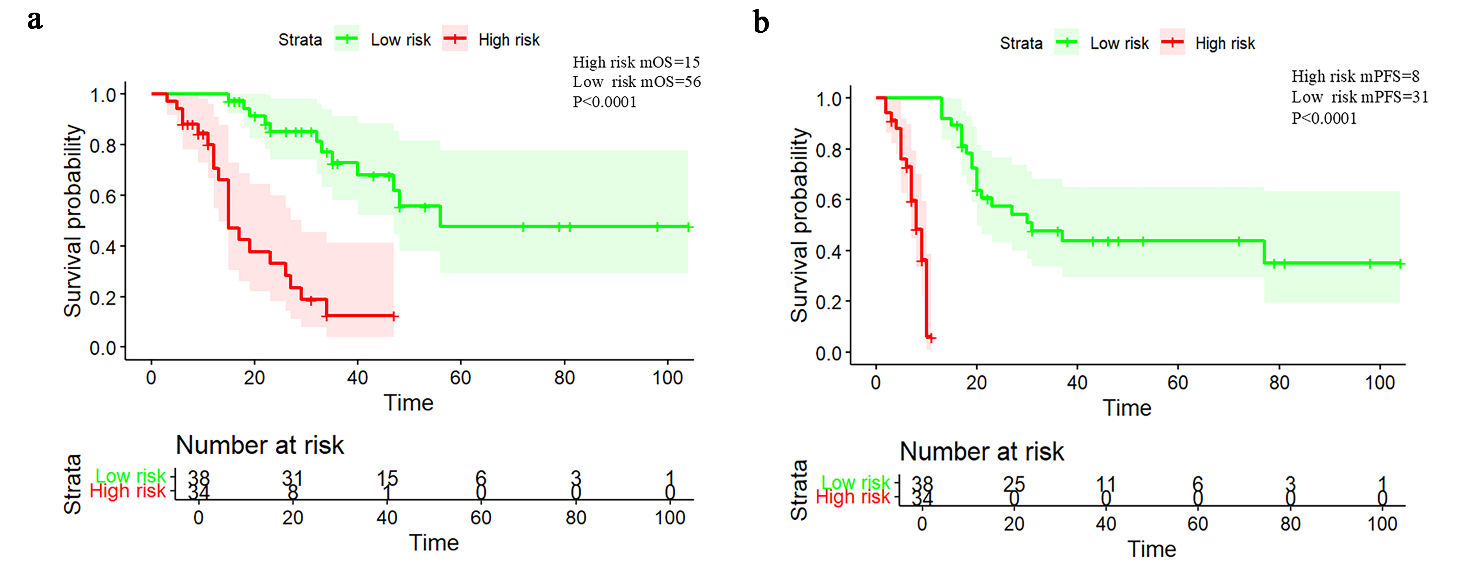
Supplementary Fig.3 Prognosis of progression groups within one year**

**Supplementary Fig.3 (a)Median PFS of progression groups within one year, (b) Median OS of progression groups within one year**

**Supplementary Eq. (A.1): K-means algorithm**

In-depth Clustering Analysis: The K-means algorithm was utilized to analyze the multidimensional feature space derived from the radiomic features. The algorithm was applied with varying numbers of cluster centers, ranging from 3 to 9, to categorize distinct habitat regions within the tumor. The performance of the clustering was evaluated using the Calinski-Harabasz score, ensuring the selection of the most statistically significant clustering arrangement.

- The K-means algorithm functions by partitioning data into K distinct clusters. It iteratively updates the centroids of these clusters to minimize the sum of squares within each cluster. The central component of the K-means algorithm is the objective function, which is optimized to achieve effective clustering.

$$\boldsymbol{J}\mathbf{=}\sum_{\boldsymbol{i}\mathbf{=1}}^{\boldsymbol{N}} \sum_{\boldsymbol{k}\mathbf{=1}}^{\boldsymbol{K}} \boldsymbol{w}_{\boldsymbol{ik}}\boldsymbol{\times\parallel}\boldsymbol{x}_{\boldsymbol{i}}\mathbf{-}\boldsymbol{\mu}_{\boldsymbol{k}}\boldsymbol{\parallel}^{\mathbf{2}}$$

- - $\boldsymbol{J}$ is the objective function.
  - $\boldsymbol{N}$ is the number of data points.
  - $\boldsymbol{K}$ is the number of clusters.
  - $\boldsymbol{w}_{\boldsymbol{ik}}$ is a binary indicator (1 if data point $\boldsymbol{i}$ is in cluster $\boldsymbol{k}$, 0 otherwise).
  - $\boldsymbol{x}_{\boldsymbol{i}}$ is the ith data point.
  - $\boldsymbol{\mu}_{\boldsymbol{k}}$ is the centroid of cluster $\boldsymbol{k}$.

$\boldsymbol{\parallel}\boldsymbol{x}_{\boldsymbol{i}}\mathbf{-}\boldsymbol{\mu}_{\boldsymbol{k}}\boldsymbol{\parallel}^{\boldsymbol{2}}$ is the squared Euclidean distance between data point $\boldsymbol{i}$ and centroid $\boldsymbol{k}$.
